# Investigation of *Bacillus cereus* growth and sporulation during *Hermetia illucens* larval rearing

**DOI:** 10.1016/j.heliyon.2024.e40912

**Published:** 2024-12-07

**Authors:** K. van Kessel, G. Castelijn, M. van der Voort, N. Meijer

**Affiliations:** Wageningen Food Safety Research, Akkermaalsbos 2, P.O. box 230, 3700 AE, Wageningen, the Netherlands

**Keywords:** *Hermetia illucens* (L.), Black soldier fly larvae (BSFL), *Bacillus cereus*, Insect rearing, Foodborne pathogens, Food safety

## Abstract

Insects are increasingly used as an alternative protein source for feed and food production. One of the main biological hazards associated with edible insects is the bio-accumulation of foodborne pathogenic microorganisms. In this study, the interaction of larvae of the black soldier fly (BSFL, *Hermetia illucens* (L.), Diptera: Stratiomyidae) with the foodborne pathogen *Bacillus cereus* was explored. As such, BSFL were reared on a substrate of wheat-based insect feed mixed with water, which was inoculated with either *B. cereus* vegetative cells or endospores*.* After seven days of rearing, the larvae and the residual substrate (frass) were analyzed for the presence of *B. cereus*, phenotypically via classical microbial counting and genotypically via real-time PCR. Endospores were detected on a selective growth medium in the larvae as well as in the frass. An additional heating step (1 min at 100 °C) to mimic blanching did not reduce the microbial count of the endospores. Results show that *B. cereus* endospores can be transferred to larvae. It is therefore recommend that substrate ingredients for BSFL rearing are tested for the presence of *B. cereus* endospores.

## Highlights

• Black soldier fly larvae were reared on substrate inoculated with *Bacillus cereus.*
• *B. cereus* endospores survived in the larvae and substrate.
• An additional heat-treatment did not kill *B. cereus* endospores.
• Vegetative cells did not survive in substrate and larvae.

## 1 Introduction

Insects have recently gained attention as an alternative nutrient source to meet the increasing demand for the world's growing population [1]. Depending on the insect species, they can enter the food chain as animal feed or directly as human food. Black soldier fly larvae (BSFL, *Hermetia illucens* (L.), Diptera: Stratiomyidae) are considered one of the species with greatest potential for feed production and are, compared to other insect species, currently produced in the largest volume for this purpose [2]. On top of that, they can be applied in the processing of organic waste. Due to the production of amylases, lipases and proteases, BSFL can convert a large variety of organic wastes, including vegetables, animal tissues and manure, into body tissue that serves as high quality nutrients for animals. In this way they can play a major role in recycling organic waste and circularizing the food chain [[2], [3], [4], [5]].

Since 2017, following the adoption of Regulation (EU) 2017/893, the use of proteins from BSFL and six other insect species, has been allowed as feed for aquaculture animals, provided that they are not reared on substrates which contain ingredients from animal origin [5,6]. From 2021 onwards, the list of authorized species has been expanded from seven to eight with the addition of silkworms. Moreover, insect proteins could also be used in feed for pigs and poultry [6,7]. Consequently, BSFL enter the feed and food chain at an increasingly broader scale [8].

As insects enter the feed and/or food chain, good hygiene and monitoring practices are needed to ensure a safe end product for both animal and human health. Specific attention needs to be paid to microbiological hazards, i.e. the growth and/or transfer of foodborne pathogenic microorganisms for animals and humans in or on the larval biomass [9]. There are several factors that may affect the dynamics of the microbiota during rearing: 1) the transfer of microorganisms from parent to offspring, 2) microorganisms that are naturally present in the rearing substrate and 3) hygiene of the rearing/production conditions [10]. When insects are exposed to foodborne pathogens during rearing, these could accumulate in the insect guts, resulting in a potential safety risk when the insects are consumed as feed or food [3,4]. The bacterial species *Bacillus* (including the *Bacillus cereus* group), *Clostridium perfringens*, *Salmonella enterica* and *Staphylococcus aureus* are the most relevant risks regarding food safety of edible insects [11,12]. Especially spore-formers, such as *Clostridium* and *Bacillus* species, are a major concern, because endospores are highly resistant to the processing stresses that are used to eliminate microbiological hazards, such as pressure sterilization [13,14].

*B. cereus* is a common cause of food poisoning in humans. Cases of infection in other mammals are more rare, but recently a large feed-related outbreak of severe infection in pigs was reported [15]. Species from the *B. cereus* group can cause two types of food-associated gastrointestinal diseases. The enteropathogenic strains cause diarrhea and abdominal pain, while the emetic types cause symptoms including nausea and vomiting. The pathogenicity of *B. cereus* is attributed to the secretion of toxins, such as hemolysin BL (Hbl), nonhemolytic enterotoxin (Nhe), cytotoxin K (CytK) and cereulide [[16], [17], [18], [19]]. Because endospores are highly resistant to (thermal) processing stresses, they can survive in the feed chain and may thereby not only endanger animal, but eventually also human health.

To explore the dynamics of microorganisms during rearing, a typical experimental design consists of inoculating the rearing substrate with the microorganism and subsequent testing for possible colonization of the substrate and insects via classical microbial counting and/or sequencing [7]. These types of inoculation experiments have been performed for a variety of food pathogens, such as *Escherichia coli*, *Staphylococcus aureus*, *Salmonella* and *Enterococcus* species [[20], [21], [22], [23], [24], [25]]. BSFL seem to exert some sort of antimicrobial activity as they seem to be capable to reduce or eliminate different food pathogens [26]. This could give the impression that the presence of food pathogens in the rearing substrate should not be a concern. There are, however, also studies that report the opposite result, namely that the number of food pathogens in the substrate stays the same or increases [7,14]. Studies on the dynamics of *B. cereus* during rearing of BSFL were lacking. Therefore, the effect of BSFL on the growth of pathogenic microorganisms has to be studied more extensively [12,14].

The aim of this study was to explore the interaction between BSFL and the foodborne pathogen *B. cereus*. The effect of BSFL on the survival and growth of *B. cereus* present in the rearing substrate was investigated, and vice versa; the effect of the *B. cereus* on the performance – in terms of survival and yield – of the BSFL. Moreover, it was investigated if the presence of *B. cereus* in the substrate causes bio-accumulation of the microorganism in BSFL. Therefore, a range of rearing trials with BSFL were conducted, after inoculating the substrate with different levels of *B. cereus* (either vegetative cells or endospores). After seven days of rearing, the presence of *B. cereus* vegetative cells or endospores in the frass (i.e. a mixture of residual feed substrate, larval feces and cuticles) and the larvae were determined.

## 2 Materials and methods

### 2.1 Experimental set-up

Different rearing conditions were included: 1) substrate without *B. cereus* and without larvae (S), 2) uninoculated substrate with larvae (S + BSFL), 3) substrate inoculated with *B. cereus* (either vegetative cells or endospores), without larvae (S + BC), 4) substrate inoculated with *B. cereus* (either vegetative cells or endospores) and with larvae (S + BC + BSFL) (Supplementary Tables 1a and b).

### 2.2 Strains and culture conditions

Two different *B. cereus* strains were used in this study: reference strain DSM31 (*hblACD* & *nheABC* positive) and strain B3465 (*nheB* & *ces* positive) originating from food (isolated within the Dutch national monitoring plan of Microbiology). Strains were stored in Brain Heart Infusion broth (BHI; Biotrading, Mijdrecht, The Netherlands) supplemented with 15 % glycerol at a temperature of −80 °C. Before use, the strains were cultivate on Tryptone Soya Agar (TSA; Biotrading, Mijdrecht, The Netherlands) for 24 ± 2 h at 30 ± 1 °C. After incubation, one single colony was suspended in 9 ml BHI broth which were statically incubated for 24 ± 2 h at 30 ± 1 °C. Subsequently, the BHI culture was enumerated by plate counts using Tryptone Soya Agar (TSA; Biotrading, Mijdrecht, The Netherlands) which were incubated 24 ± 2 h at 30 ± 1 °C.

Spores were obtained by inoculating colony material from TSA on Hydrolysate of Casein Tryptone (HCT) agar medium (preparation in accordance with ISO 7932/A1:2020) at 30 ± 1 °C for 5 days, at which >90 % of the culture consisted of free spores (examined by phase contrast microscopy). Spores were harvested (based on the method as described by Ceupens et al. [27]) by dissolving colony material in 20 mL sterile physiological salt solution (0.85 % NaCl). After centrifugation at 10,000×*g* for 15 min the spore pellet was washed with 10 mL sterile physiological salt solution. This washing step was repeated once, but then the pellet was dissolved in 10 mL of ethanol (50 %) solution. Then, the spore suspension was incubated at 5 ± 3 °C overnight (18 h) to eliminate vegetative cells. The washing procedure was repeated twice. Finally, the endospores were suspended in 10 mL sterile distilled water and kept at 5 ± 3 °C until further use. The exact spore concentration was determined by plating appropriate dilutions on TSA.

### 2.3 Substrate inoculation

Substrate was prepared by mixing the finely ground wheat bran (Meelfabriek De Jongh, Steenwijk, The Netherlands) and tap water in a 35:65 ratio (w/w). 50 g of the substrate was inoculated with vegetative cells or endospores with target levels of 4, 6 and 8 log CFUs. The added inoculum volume was 1 mL, so a final ratio of 35:65 (w/w) was obtained. To the uninoculated rearing conditions (S and S + BSFL), 1 mL tap water was added to obtain the same ratio. Both the inoculated and uninoculated substrates were homogenized by stirring and left at room temperature. Added concentrations can be found in Supplementary Table 1a.

The different rearing conditions S, S + BSFL and S + BC + BSFL were performed in triplicate. Rearing condition S + BC was performed in duplicate.

50 mL of BHI contaminated in duplicate with the lowest spike concentration (4 log CFUs) were included as additional control to test the inoculum. To exclude contamination of the BHI, a blank BHI control (50 mL) were included in duplicate. (Supplementary Table 1b).

### 2.4 BSFL rearing and sampling

BSFL originated from a colony maintained by InsectoCycle (InsectoCycle; Wageningen; The Netherlands). During the first 7 days, larvae were grown on standard feed, containing wheat, cornmeal, soy scrap Hipro, brewer's yeast and barley at 27–29 °C, with a relative humidity of 55 %.

At day 7, an intended number of 50 larvae were added to each relevant cultivation tray containing 50 g of diet, i.e. 1 larva/gram of diet (S + BSFL and S + BC + BSFL). The cultivation trays were cylindrical (diameter 100 mm, height 40 mm) and were closed with a lid containing a circular area (diameter 40 mm) in the center which was covered by a mesh (SPL Life Sciences Co., Ltd., Gyeonggi-do, South Korea) to allow air circulation, but to prevent the escape of larvae. Before the start of the experiment, it was verified whether the larvae were not already naturally infected with *B. cereus*, as described in sections 2.6, 2.7.

The larvae were reared for 7 days until day 14 post-hatching, in a climate chamber at 28 °C. The samples without larvae were incubated under the same conditions. Sampling of larvae and frass took place on day 7. Larvae were separated from the substrate using a sterile tweezer and then washed twice in sterile, demineralized water to remove adhering residual material. Finally, the larvae were collected and placed in sterile plastic bags. The larvae were then killed by pulverizing them within the plastics bags, utilizing mechanical pressure (rolling pin) to ensure immediate death. Subsequently, frass and larvae samples were weighed, transferred in a sterile filter bag and diluted ten-fold in Peptone Physiological Salt Solution (PPS; Biotrading, Mijdrecht, The Netherlands). Larval and frass were homogenized for 60 s using a Smasher (bioMérieux).

### 2.5 Heat-treatment

1 mL of the homogeneous suspension in PPS (section 2.4) was transferred to a sterile 1.5 mL Eppendorf tube. The samples were heated at 100 °C for 1 min in a thermoblock (Eppendorf ThermoMixer F1.5, Hamburg, Germany) at 800 rpm, to simulate the post-harvesting processes (blanching).

To check the effect of the heat-treatment on vegetative cells and to check whether spores were present in the vegetative inoculum, an additional experiment was conducted. For this, both strains were grown in triplicate in BHI for 24 ± 2 h at 30 ± 1 °C. One replicate was heated at 100 °C for 1 min, as used in the original experiment. The second replicate was heated at 80 °C for 10 min (pasteurization, to eliminate vegetative cells) (NEN 6813:2014). And the last replicate was taken untreated. The different samples were enumerated by plate counts using TSA which were incubated 24 ± 2 h at 30 ± 1 °C.

### 2.6 Microbiological analyses

Larvae and frass from both the untreated and heat-treated samples were analyzed for the presence of presumptive *B. cereus*, by using 100 μL of tenfold dilution series for plate counts on BACARA® agar (bioMérieux Benelux B.V.; Amersfoort; The Netherlands) at 37 ± 1 °C for 24 ± 2 h. Presumptive colonies randomly selected and confirmed on the Microflex LT/SH™ mass spectrometer (Bruker Daltonics GmbH & Co. KG; Bremen; Germany) by the direct colony method [28].

### 2.7 Confirmation by real-time PCR

Presence of *B. cereus* in both the untreated and heat-treated samples was analyzed by real-time PCR for presence of the enterotoxin gene *nheB*, as this gene is present in both strains used. For this purpose the untreated and heat-treated samples were diluted tenfold in PPS and DNA was extracted by using bead-beating followed by DNA purification. 500 μL of the sample was transferred to a new 1.5 mL Eppendorf tube. After centrifugation at 10,000×*g* for 15 min, the pellet was dissolved in 500 μL ZymoBIOMICS Lysis Solution (Zymo Research Europe; Freiburg im Breisgau; Germany) and transferred to a 2 mL tube containing 0.1 mm silica beads (MP Biomedicals; Eschwege; Germany). Bead-beating was performed in a Fastprep-24™ device (MP Biomedicals) in five cycles of 1 min (6.5 m/s), with a 5 min period of rest at room temperature. The tubes were centrifuged at 10,000×*g* for 1 min and 75 μL of the supernatant was used for further purification. This was performed with the KingFisher Flex Purification System (Thermo Fisher Scientific, Breda, The Netherlands) using the QuickPick Plant DNA kit (Bio-Nobile, Pargas, Finland) according to manufacturer's instructions.

Detection of the representative toxin gene *nheB* was based on real-time PCR using the following nucleotides: forward primer (nheB-FW1) 5′-GCAGCTGAAAGTACAGTGAAAC-3′, reverse primer (nheB-RV1) 5′-TCAAGCCTTCTGGTCCTAATG-3′ and probe (nheB-P1) 5′-HEX-CGCCAGTTCATGCGGTAGCAAA-BHQ1-3’. The primers were designed based on the *nheABC* gene sequence (NCBI accession number Y19005.2 (861.2021bp)). As internal amplification control (IAC) the primers and probe described by Deer et al. [29] were used. Each 25 μL reaction volume contained 12.5 μL 2x TaqMan Multiplex Master mix, 150 nM IAC-probe, 200 nM IAC-forward primer/IAC-reverse primer/nheB-forward primer, 300 nM nheB-probe, 400 nM nheB-reverse primer and 3 μL DNA template. The amplification program, carried out on a CFX96 Touch Real-Time PCR Detection System (Bio-Rad Laboratories B.V.; Veenendaal; The Netherlands), consisted of an initial denaturation at 95 °C for 15 min, followed by 40 cycles at 95 °C for 10 s for denaturation and 58.5 °C for 60 s for primer binding and extension. For PCR results, the increase in the fluorescence signal of the reporter dye detected was visualized by the CFX Maestro software v2.3 (Bio-Rad). Quantification cycle (Cq) values represent the PCR cycle in which a first increase in fluorescence over a defined threshold occurred for each amplification plot.

### 2.8 Statistical analysis

For statistical analyses, the software SPSS Statistics for Microsoft Windows (version 25.0.0.2, IBM Corp., Armonk, NY, United States) was used. Because the treatments were performed in triplicate, a Gaussian distribution could not be assumed. Therefore, non-parametric statistical tests were performed to determine the statistical significance of the results. Statistical analysis was performed on the S + BC + BSFL conditions: larvae vs frass and untreated vs heat-treated samples. The distributions of the treatments were compared using a Kruskal-Wallis test (α = 0.05).

## 3 Results

### 3.1 Control experiments

In the inoculated samples without larvae (BHI medium + BC and S + BC), both strains (vegetative cells and endospores) could be detected, confirming that the *B. cereus* cultures were viable and able to colonize the substrate (Supplementary Tables 1b and 2). As expected, the uninoculated substrates without and with larvae (S and S + BSLF) were below the detection limit, which shows that the larvae were not naturally infected with *B. cereus* (Supplementary Tables 1b and 2).

In addition to analyzing the samples via enumeration on BACARA plates, their DNA was extracted. The isolated DNA was analyzed by real-time PCR for the presence of *B. cereus*-specific enterotoxin gene *nheB*. The Cq-values are an indication for the amount of *nheB* (and thereby *B. cereus*) present in the sample. The obtained Cq-values show that both the inoculum of strain B3465 and of strain DSM31 contained *nheB* at a high level (Cq-value of respectively 20 and 18 on average), confirming the results that were obtained by plate counting (Supplementary Table 2). The Cq values from the BHI medium + BC and/or S + BC condition give an indication for the *B. cereus* levels that could be found in the larvae and frass.

### 3.2 Inoculation of rearing substrates

Dilution ranges of the *B. cereus* strains were prepared in order to inoculate the substrates with target levels of 8, 6 and 4 log CFUs/50 g substrate, which equals 7.3, 5.3 and 3.3 log CFUs/gram. The actual obtained concentrations were close to the target levels, namely: for B3465 vegetative cells 5.20, 3.20 and 1.20; for B3465 spores 6.05, 4.05 and 2.05; for DSM31 vegetative cells 7.19, 5.19 and 3.19; and for DSM31 spores 7.06, 5.06 and 3.06 log CFUs/gram substrate (Supplementary Table 1a). These 12 inoculated substrates were used for rearing experiments, all conditions were tested in triplicate.

### 3.3 Rearing of BSFL

**Fig. 1:**
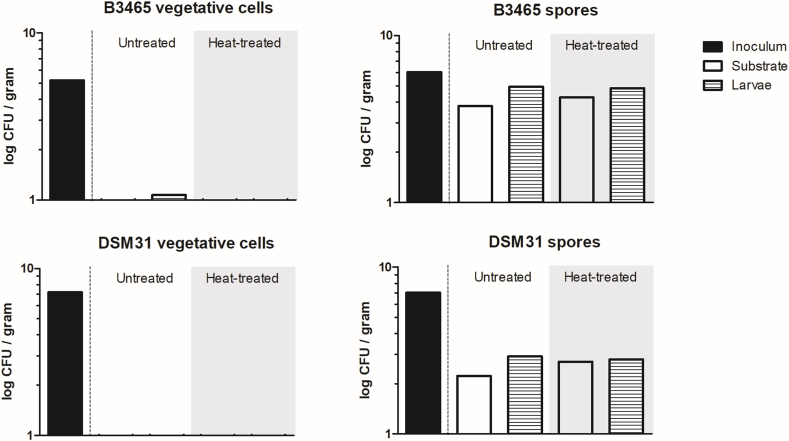
Presence of B. cereus vegetative cells and endospores in larvae and residual substrate after 7 days of BSFL rearing. Graphs represent B. cereus counts in log CFU/gram sample for B3465 (upper graphs) and DSM31 (lower graphs), vegetative cells (left) and endospores (right), highest inoculation levels. Black bars indicate the inoculation levels, white bars the residual substrates and striped bars the larvae. Grey-shaded results indicate the heat-treated samples. Kruskal-Wallis test (α = 0.05), n.s.

BSFL were reared on these substrates for seven days, after which the frass and the larvae were analyzed for the presence of *B. cereus* by plate counts on BACARA. In the conditions where the substrates were inoculated with vegetative cells, *B. cereus* could not or barely be detected in the larvae after seven days of rearing (Table 1, Fig. 1). This could indicate that vegetative cells were not transferred to the larvae or that the vegetative cells were initially transferred to the larvae during the first days of rearing, but were eliminated in their body due to antimicrobial activity. Comparing the results of the frass with the larvae-free controls (S + BC condition), the detectable levels of *B. cereus* were lower. However, it must be pointed out here that the detectable levels in the larvae-free controls were already quite low, shown by high Cq values (Supplementary Table 2).

**Tabel 1:** Enumeration of typical B. cereus on BACARA plates (expressed in log CFU/gram; average of biological triplicates) and Cq values of real-time PCR for the detection of nheB – B3465 and DSM31 vegetative cells. Arithmetic mean and standard deviation. Light-grey shaded: heat-treated samples (1 min at 100 °C). Kruskal-Wallis test (α = 0.05), n.s.

	B3465 vegetative cells								
Experimental condition	Inoculation level (log CFU/gram)	Frass				Larvae			
		Unprocessed		Heated		Unprocessed		Heated	
		Count (log CFU/gram)	Cq (qPCR)	Count (log CFU/gram)	Cq (qPCR)	Count (log CFU/gram)	Cq (qPCR)	Count (log CFU/gram)	Cq (qPCR)
S + BC + BSFL	5.20	0.23 ± 0.41	N/A N/A N/A	0.33 ± 0.58	N/A N/A 39.62	0.52 ± 0.91	N/A N/A N/A	<0	N/A N/A N/A
	3.20	0.21 ± 0.37	N/A 38.18 N/A	<0	32.73 36.83 N/A	<0	N/A 39.89 N/A	0.32 ± 0.56	N/A 38.15 N/A
	1.20	<0	N/A N/A N/A	<0	37.41 N/A N/A	<0	N/A N/A N/A	<0	N/A N/A N/A
DSM31 vegetative cells									
S + BC + BSFL	7.19	<0	N/A N/A N/A	<0	37.87 N/A N/A	<0	N/A N/A N/A	<0	N/A N/A N/A
	5.19	<0	N/A N/A N/A	<0	N/A N/A N/A	<0	N/A N/A N/A	<0	N/A N/A N/A
	3.19	<0	N/A N/A N/A	<0	N/A N/A N/A	<0	N/A N/A N/A	<0	N/A N/A N/A

In the conditions where the rearing substrates were inoculated with endospores, *B. cereus* was detected in the frass as well as in the larvae after seven days of rearing. The *B. cereus* load detected in the larvae was equally high as in the substrate.

Though the spike concentrations of strain B3465 were lower than of strain DSM31, inoculation of the rearing substrate with B3465 endospores resulted in equal (or even trending towards higher) microbial loads in the frass and larvae than inoculation with strain DSM31 (Table 2) (Fig. 1), suggesting that the survival properties of *B. cereus* are strain-dependent.

### 3.4 Effect of heat-treatment on the presence of B. cereus

The inoculated substrates were heat-treated and tested for the presence of *B. cereus*. Surprisingly, CFUs were found for substrates that were inoculated with vegetative cells (Supplementary Table 2). This suggested that spores might also have been preset in the vegetative inoculum. Therefore, overnight cultures from the two *B. cereus* strains were exposed to the heat-treatment used in this study to mimic blanching (1 min at 100 °C) and a standard treatment used to eliminate vegetative cells (10 min at 80 °C) (Supplementary Table 3). Since both heat-treatments equally reduced the cell count, the results indicated that indeed spores were present in the culture of vegetative cells. Hence, it is concluded that the CFUs that were detected in the substrate upon heat-treatment (Supplementary Table 2) are thus formed by the spores that were present in the culture of vegetative cells, and not from vegetative cells. Remarkably, this reduction in CFU counts upon heating on the DSM31 culture was more pronounced than on the B3465 culture, indicating that the percentage of spores in the B3465 culture was considerably higher.

In the inoculated substrates with larvae (S + BC + BSFL) the additional heating step did not make a difference when the substrate was inoculated with vegetative cells, since there were few *B. cereus* cells present to kill in the first place (Table 1). The small amount of cells that are detected must be the endospores that were present in the inoculum, because they are not killed by the heating step. In the conditions where the rearing substrates were inoculated with spores, an additional heating step did not reduce the amount of CFUs detected (Table 2, highest inoculation levels are depicted in Fig. 1). The additional heating step considerably reduced the amount of background microbiota (while the effect on *B. cereus* CFUs is minimal as observed in the case of larvae), enabling counting of *B. cereus* CFUs. Data obtained by real-time PCR confirmed that there were approximately equal levels of *nheB* present in the frass as in the larvae (Table 2). In the cases where the rearing substrate was inoculated with a lower spike concentration, the Cq-values were higher, indicating a lower level of the *nheB* gene.

**Table 2:** Enumeration of typical B. cereus on BACARA plates (expressed in log CFU/gram; average of biological triplicates) and Cq values of real-time PCR for the detection of nheB – B3465 and DSM31 spores. Arithmetic mean and standard deviation Light-grey shaded: heat-treated samples (1 min at 100 °C). Kruskal-Wallis test (α = 0.05), n.s.

	B3465 spores								
Experimental condition	Inoculation level (log CFU/gram)	Frass				Larvae			
		Unprocessed		Heated		Unprocessed		Heated	
		Count (log CFU/gram)	Cq (qPCR)	Count (log CFU/gram)	Cq (qPCR)	Count (log CFU/gram)	Cq (qPCR)	Count (log CFU/gram)	Cq (qPCR)
S + BC + BSFL	6.05	3.78 ± 0.08	31.53 32.02 31.57	4.25 ± 0.14	30.89 31.11 30.26	4.93 ± 0.04	30.39 30.70 30.30	4.83 ± 0.15	30.05 30.19 29.89
	4.05	0.84 ± 0.78	N/A 39.54 N/A	1.56 ± 0.35	38.20 N/A N/A	2.26 ± 0.09	38.53 37.86 N/A	2.51 ± 0.10	38.29 37.02 38.17
	2.05	<0	N/A N/A N/A	<0	39.60 N/A N/A	<0	N/A N/A N/A	0.65 ± 0.57	N/A N/A N/A
DSM31 spores									
S + BC + BSFL	7.06	1.75 ± 1.57	31.72 32.39 30.63	2.71 ± 0.11	31.25 32.29 30.57	3.03 ± 0.18	32.87 33.34 30.53	2.78 ± 0.16	31.59 32.87 30.98
	5.06	0.35 ± 0.61	38.32 39.62 35.15	1.11 ± 0.50	38.37 37.70 36.67	0.43 ± 0.75	36.31 38.22 N/A	0.33 ± 0.58	39.52 37.58 37.49
	3.06	<0	N/A N/A 39.73	<0	N/A N/A N/A	<0	N/A 39.25 N/A	0.33 ± 0.56	N/A N/A N/A

## 4 Discussion

To investigate the dynamics of the foodborne pathogen *B. cereus* during rearing BSFL, rearing substrate was inoculated with either vegetative cells or endospores. *B. cereus* endospores were found to survive in BSFL and in the frass, while vegetative cells did not survive, neither in BSFL nor in the substrate.

Regulation (EC) No 142/2011 requires that for the production of animal feed, processed animal proteins (PAPs) of non-mammalian origin must have been processed in accordance with one of five standard methods to reduce microbiological contamination (Chapter 3 of Annex 4). Depending on the particle size; certain time, temperature, and pressure requirements are prescribed. Alternatively, ‘method 7’ involves authorization of a novel method by the respective competent national authority, which requires demonstration of reduction of *Clostridium perfringens*, *Salmonella*, and *Enterobacteriaceae* – but not *B. cereus*. Based on these observations, it is hypothesized that blanching may reduce the *B. cereus* vegetative cell count, but does not kill endospores and therefore does not completely sterilize the sample. In this way, endospores could end up in the feed chain and potentially endanger animal and eventually human health.

Remarkably, the data suggests that the presence of BSFL reduces vegetative *B. cereus* growth, because in the S + BC + BSFL condition, *B. cereus* could not be detected in the larvae nor in the frass, while the level in the larvae-free controls (S + BC) was higher. In the case of absence of vegetative *B. cereus* – or even reduction as observed in this study – formation of toxins is unlikely. However, the presence of toxins was not tested, so it cannot be ruled out that this could still pose a safety problem if formed prior to the insect rearing stage. A possible explanation for the observation that the presence of larvae reduced the level of detected *B. cereus*, is the excretion of antimicrobial peptides by BSFL. Previous studies, in which these type of inoculation experiments have been performed for *Escherichia coli*, *Staphylococcus aureus*, *Salmonella* species and *Enterococcus* species [[20], [21], [22], [23], [24], [25]], reported antimicrobial activity of BSFL as they are capable of suppressing the growth of different microorganisms by the secretion of antimicrobial compounds [26]. Another possible explanation is that spores germinate and that, subsequently, these vegetative bacteria do not survive. The finding that the detectable levels of *B. cereus* are almost equally high before and after heat-treatment, indicates that the detected *B. cereus* are endospores, either formed during the rearing experiment, or because they were already present in the inoculum. To get a clearer view on the dynamics of *B. cereus* vegetative cells during insect rearing more investigation is required, especially by measuring on different time points during the rearing experiment.

If the presence of BSFL reduces *B. cereus*, that could give the impression that the presence of food pathogens in the rearing substrate should not be a concern. However, a recent study by Moyet et al. [14] reported that the presence of BSFL in potato substrate increased the survival and growth of *B. cereus*, and a study by De Smet et al. [7] the increase of *Salmonella*. These contradicting observations suggest that the antimicrobial activity may be bacterial species and substrate dependent [26]. To what extent the findings of this study can be extrapolated to other substrates, such as from waste-streams, should therefore be subject of further research.

In the current study, the endogenous microbiota present in the feed were also active during the experiments: the wetted substrate stored at 28 °C formed an ideal environment for the growth of background microorganisms, especially fungi. In initial pilot experiments, growth of background fungi hindered the counting of *B. cereus* colonies on Mannitol egg Yolk Polymyxin (MYP) plates. Previous studies also reported hinder of background microbiota in the rearing substrates and advised the use of an extra selective or elective aid (such as the introduction of an antibiotic-resistance gene in the target microorganism) [7,14]. Therefore, natamycin was added as an anti-fungal compound to the MYP plates. This indeed inhibited the growth of fungi, but did not hinder the growth of other bacteria (data not shown), which were still hindering an accurate count of *B. cereus* CFUs. It was therefore decided to plate the samples on BACARA agar, which is a chromogenic medium that is selective for *B. cereus* species. In some of the conditions tested, there still grew many background microbiota on the BACARA plates, indicating that they must be closely related to *B. cereus*. Further diluting the samples also diluted the background microbiota and made it possible to count the *B. cereus* CFUs. Though the background microbiota did in this way not influence the microbiological analysis, any inhibiting or competing effects of the background microbiota on the growth of *B. cereus* in the substrate cannot be ruled out. Results from these pilot experiments also suggested that vegetative *B. cereus* inoculated at low levels did not survive in the substrate, hence selecting comparatively high inoculation levels to ensure analytical recovery and mimic a commercial worst-case scenario.

This study shows that *B. cereus* endospores present in the rearing substrate, can be transmitted from substrate to black soldier fly larvae and that the heating step tested in this study did not reduce endospore count in the larvae. As a consequence, endospores may end up in the feed chain that pose a safety hazard to animals and humans. Consequently, to avoid *B. cereus* endospores from entering the feed and food chain and to ensure a safe end product for both animals and humans, it is advised to test substrate ingredients for the presence of *B. cereus* for BSFL rearing. According to Dutch Regulation BWBR0005758, the level of *B. cereus* per gram/mL of food stuff, should be lower than 5 log CFUs. In France, the same threshold is applied [30]. It is suggested that insect producers maintain this norm.

## 5 Conclusion

In this study it was shown that *B. cereus* endospores present in the rearing substrate were transferred to the larvae of the black soldier fly. The endospores also survived in the frass and their counts were not reduced by potential antimicrobial activity of the larvae. *B. cereus* vegetative cells were below the detection limit, but it could not be proven that the pathogen was not initially ingested and eliminated during the rearing experiment, as the vegetative bacteria did not survive either in the substrate without larvae. An additional heat-treatment did not kill the endospores. Furthermore, the microbial load detected in the larvae and frass was strain-dependent. In conclusion, to ensure a safe end product for animal and human health, it is recommended to analyze substrate ingredients for the absence of *B. cereus* spores.

## CRediT authorship contribution statement

**K. van Kessel:** Writing – original draft, Methodology, Investigation, Data curation. **G. Castelijn:** Writing – review & editing, Supervision, Conceptualization. **M. van der Voort:** Writing – review & editing, Supervision, Conceptualization. **N. Meijer:** Writing – review & editing, Methodology, Funding acquisition, Conceptualization.

## Data availability statement

Relevant data from the experiments is provided in the manuscript or supplementary materials. Any other data can be made available upon request, depending on confidentiality.

## Funding statement and conflicts of interest

This project was funded by the Dutch Ministry of Economic Affairs through a Public-Private Partnership project (“Controlling the safety of insects for food and feed”) of the Topsector AgriFood (LWV19099). The Ministry had no role in study design, data collection and analysis, decision to publish, or preparation of the manuscript. The commercial entities within the project consortium were: Proti-Farm R&D, BV; Protix Biosystems; Bestico B.V., and ForFarmers. The commercial entities in the consortium had no role in study design, data collection and analysis, decision to publish, or preparation of the manuscript.

## Declaration of competing interest

The authors declare the following financial interests/personal relationships which may be considered as potential competing interests: Nathan Meijer reports financial support was provided by Dutch Ministry of Economic Affairs. This project was funded by the Dutch 10.13039/501100004725Ministry of Economic Affairs through a Public-Private Partnership project (“Controlling the safety of insects for food and feed”) of the Topsector AgriFood (LWV19099). The Ministry had no role in study design, data collection and analysis, decision to publish, or preparation of the manuscript. The commercial entities within the project consortium were: Proti-Farm R&D, BV; Protix Biosystems; Bestico B.V., and ForFarmers. The commercial entities in the consortium had no role in study design, data collection and analysis, decision to publish, or preparation of the manuscript. If there are other authors, they declare that they have no known competing financial interests or personal relationships that could have appeared to influence the work reported in this paper.
