# Supplementary material for: Investigation of Bacillus cereus growth and sporulation during Hermetia illucens larval rearing
Source: Heliyon. 2024 Dec 7;10(24):e40912. doi: 10.1016/j.heliyon.2024.e40912 (PMC11696645; doi:10.1016/j.heliyon.2024.e40912)
Supplement: Multimedia component 1 [file mmc1.docx]

Supplementary table 1a: Set-up of the rearing experiments and the inoculation levels of the biological triplicates of B3465 and DSM31 vegetative cultures and spore suspensions. ‘H’ indicates heat treatment for 1 min at 100 °C; ‘-’ indicates no additional processing steps were performed.

| Experimental condition | *Bacillus cereus*  strain | Vegetative or  spores | Inoculation level  (log CFU / 50 gram) | Inoculation level  (log CFU / gram) | Processing |
| --- | --- | --- | --- | --- | --- |
| S + BC + BSFL | B3465 | Vegetative cells | 6.90 | 5.20 | - |
|  |  |  |  |  | H |
|  |  |  | 4.90 | 3.20 | - |
|  |  |  |  |  | H |
|  |  |  | 2.90 | 1.20 | - |
|  |  |  |  |  | H |
|  |  | Spores | 7.75 | 6.05 | - |
|  |  |  |  |  | H |
|  |  |  | 5.75 | 4.05 | - |
|  |  |  |  |  | H |
|  |  |  | 3.75 | 2.05 | - |
|  |  |  |  |  | H |
| S + BC + BSFL | DSM31 | Vegetative cells | 8.89 | 7.19 | - |
|  |  |  |  |  | H |
|  |  |  | 6.89 | 5.19 | - |
|  |  |  |  |  | H |
|  |  |  | 4.89 | 3.19 | - |
|  |  |  |  |  | H |
|  |  | Spores | 8.76 | 7.06 | - |
|  |  |  |  |  | H |
|  |  |  | 6.76 | 5.06 | - |
|  |  |  |  |  | H |
|  |  |  | 4.76 | 3.06 | - |
|  |  |  |  |  | H |

Supplementary table 1b: Set-up of control experiments.

| Experimental  condition | *Bacillus cereus* strain | Vegetative or  spores | Inoculation level  (log CFU / 50 gram) | Processing | Number of replicates |
| --- | --- | --- | --- | --- | --- |
| BHI medium | - | - | - | - | 2 |
| BHI medium + BC | B3465 | Vegetative cells | 4 | - | 2 |
|  |  | Spores | 4 | - | 2 |
|  | DSM31 | Vegetative cells | 4 | - | 2 |
|  |  | Spores | 4 | - | 2 |
| S + BC | B3465 | Vegetative cells | 8 | - | 2 |
|  |  |  |  | H | 2 |
|  |  | Spores | 8 | - | 2 |
|  |  |  |  | H | 2 |
|  | DSM31 | Vegetative cells | 8 | - | 2 |
|  |  |  |  | H | 2 |
|  |  | Spores | 8 | - | 2 |
|  |  |  |  | H | 2 |
| S | - | - | - | - | 3 |
| S + BSFL | - | - | - | - | 3 |

Supplementary table 2: Enumeration of typical B. cereus on BACARA plates (undiluted and 100x diluted) and Cq values of real-time PCR for the detection of nheB – control experiments. All conditions were performed in biological duplicate. Light-grey shaded: heat-treated samples (1 min at 100 °C); dark-grey shaded: not applicable or not performed. N.d.: not detected. Kruskal-Wallis test (α=0.05), n.s.

|  |  |  | Control experiments | | | | | | | | |
| --- | --- | --- | --- | --- | --- | --- | --- | --- | --- | --- | --- |
| Experimental  condition | *Bacillus cereus* strain | Vegetative or endospores | Inoculation level  (log CFU / 50 gram) | Substrate | | | | | | Larvae | |
|  |  |  |  | Unprocessed | |  | Heated | | | Unprocessed | |
|  |  |  |  | Count (culture) | | Cq (qPCR) | Count (culture) | | Cq (qPCR) | Count (culture) | Cq (qPCR) |
|  |  |  |  | Undiluted | Diluted 100x |  | Undiluted | Diluted 100x |  | Undiluted |  |
| BHI medium + BC | B3465 | Vegetative | 4 | >150 | >150 | 19.82 |  |  |  |  |  |
|  |  |  |  | >150 | >150 | 20.14 |  |  |  |  |  |
|  |  | Spores | 4 | >150 | >150 | 20.17 |  |  |  |  |  |
|  |  |  |  | >150 | >150 | 20.49 |  |  |  |  |  |
|  | DSM31 | Vegetative | 4 | >150 | >150 | 18.70 |  |  |  |  |  |
|  |  |  |  | >150 | >150 | 18.09 |  |  |  |  |  |
|  |  | Spores | 4 | >150 | >150 | 18.99 |  |  |  |  |  |
|  |  |  |  | >150 | >150 | 18.38 |  |  |  |  |  |
| S + BC | B3465 | Vegetative | 8 | >150 |  | 33.77 | >150 |  | 34.65 |  |  |
|  |  |  |  | >150 |  | 33.95 | >150 |  | 35.43 |  |  |
|  |  | Spores | 8 | >150 | 69 | 30.31 | >150 | 28 | 31.28 |  |  |
|  |  |  |  | >150 | 37 | 31.23 | >150 | 64 | 31.88 |  |  |
| S + BC | DSM31 | Vegetative | 8 | 58 |  | 32.35 | 128 |  | 33.11 |  |  |
|  |  | Spores | 8 | >150 | >150 | 27.42 | >150 | 86 | 27.85 |  |  |
|  |  |  |  | >150 | 112 | 27.05 | >150 | 62 | 27.76 |  |  |
| S |  |  |  | 0 |  | n.d. |  |  |  |  |  |
|  |  |  |  | 0 |  | n.d. |  |  |  |  |  |
| S + BSFL |  |  |  | 0 |  | n.d. |  |  |  | 0 | n.d. |
|  |  |  |  | 0 |  | n.d. |  |  |  | 0 | n.d. |
|  |  |  |  | 0 |  | n.d. |  |  |  | 0 | n.d. |

Supplementary table 3: Comparison of heat-treatments (untreated; 1 min at 100 °C and 10 min at 80 °C) on the enumeration of typical B. cereus on BACARA plates. Results show the B. cereus concentration of the vegetative cell suspension (strain B3465 and strain DSM31) in CFU/mL. Arithmetic mean and standard deviation.

|  | B3465 | DSM31 |
| --- | --- | --- |
| Experimental  condition | log CFU/mL | log CFU/mL |
| Untreated | 7.75 ± 0.33 | 7.37 ± 0.04 |
| Heated 1 min @ 100°C | 6.24 ± 0.89 | 4.31 ± 0.30 |
| Heated 10 min @ 80°C | 6.04 ± 0.93 | 4.18 ± 0.39 |
